# Alpha1-Antitrypsin in Lung Diseases: A Cross-Sectional Observational Study

**DOI:** 10.3390/ijms26115400

**Published:** 2025-06-04

**Authors:** Csilla Páska, Imre Barta, Zsuzsanna Csoma, Réka Gajdócsi, Viktória Szél, Anna Kerpel-Fronius, Diána Solymosi, Zoltán Örlős, Balázs Antus

**Affiliations:** National Korányi Institute for Pulmonology, Korányi Frigyes út. 1, 1121 Budapest, Hungary

**Keywords:** antitrypsin, lung pathologies, COPD, asthma, ILD, sarcoidosis, cystic fibrosis

## Abstract

Major mutations of *SERPINA1*, the gene encoding alpha1-antitrypsin (A1AT), are known to cause severe emphysema. Our study aimed to investigate the role of major mutations modulating A1AT levels in several lung pathologies and control groups. Blood samples were collected from healthy non-smokers (N^0^ = 85), healthy smokers (N^0^ = 291), healthy ex-smokers (N^0^ = 127), smokers with chronic obstructive lung disease (COPD, N^0^ = 187), ex-smokers with COPD (N^0^ = 64), and patients with asthma (N^0^ = 194), interstitial lung disease (ILD) (N^0^ = 93), sarcoidosis (N^0^ = 30) and cystic fibrosis (N^0^ = 26). Clinical and respiratory parameters, A1AT levels, the extent of emphysema and comorbidities on low-dose CT scans were evaluated, and patients answered a smoking history and comorbidity questionnaire. A1AT single-nucleotide polymorphisms were determined for the S, Z, M2/M4, 0 and eQTL locations by SNP probes using real-time PCR. A1AT levels showed significant differences between cigarette smoke-induced and other lung diseases. Compared to controls, A1AT levels were found to be lower in sarcoidosis and increasingly higher in smokers and patients with COPD, ILD and CF, respectively. The presence and pattern of emphysema were found to influence A1AT levels: lower values were observed in COPD patients without emphysema, while higher values were observed in patients with central and panlobular emphysema. Antitrypsin levels increased with COPD GOLD stages and asthma GINA stages. Variable A1AT levels were also found in ILD subgroups. The distribution of variants at the S, Z, M2/M4 and 0 polymorphic sites and the eQTL location showed no significant differences between patient groups with impaired lung function, except for Z heterozygotes, which were prevalent in patients with severe asthma. The eQTL TT genotypes had higher A1AT levels and the occurrence of emphysema and/or bronchitis was increased. A1AT levels correlated with several clinical and respiratory parameters in pulmonary patients, while FEV1/FVC inversely correlated with levels of A1AT. Molar antielastase activity was increased in smokers and patients with lung diseases; however, in COPD, antielastase activity decreased. The most reduced antielastase activity could be found in CF. Certain genotypes were characterized by increased cardiovascular comorbidity scores and antitrypsin levels. Our data suggest that in addition to emphysema, A1AT may play an important role in the development of a wide variety of lung diseases and cardiovascular comorbidities. Further research is needed to clarify the role of A1AT and its regulation in lung pathologies.

## 1. Introduction

Mutations in the gene coding for alpha1-antitrypsin (A1AT) have long been a focus of chronic obstructive lung disease (COPD) research as a causative factor of early severe emphysema. In addition to its antielastase activity, the A1AT molecule has a surprisingly broad function, which is explained by the conformational polymorphism of the A1AT protein [1,2]. In these alternative functions, A1AT adopts conformations other than those required for blocking neutrophil elastase [3]. Moreover, A1AT is also an antioxidant involved in anti-inflammatory processes, and its levels are elevated in inflammatory diseases [4,5,6]. The baseline level and the release rate of A1AT are important in the acute-phase reaction and its resolution [1,2].

Based on these functions, A1AT was thought to play a protective role in lung injury [7,8,9]. The immunomodulatory properties of A1AT are determined by its glycoside side chains [10,11,12]. The multiple isoforms of A1AT differ in the post-translational glycan modifications at Asn70, Asn107 and Asn271 [13], and smoking induces changes in glycosylation patterns [14,15]. With the loss of its lipid-binding capacity that confers a protective effect [2,16,17,18], A1AT is implicated in the development of metabolic syndrome and atherosclerosis as major comorbidities commonly found in smokers. Cigarette smoke-induced oxidation leads to alterations in some A1AT functions, notably elastase activity, while others are unaffected [19,20]. These additional functions may also play an important role in the development of COPD and its comorbidities.

Antitrypsin, an acute-phase protein capable of cleaving many proteases [21], is synthesized mainly in the liver and, to a lesser extent, in neutrophils, monocytes and lung epithelial cells [22] before being released into the serum. The gene encoding antitrypsin, *SERPINA1*, is located on chromosome 14q32.1 and consists of four coding exons, three untranslated exons and six introns. Its structure is composed of three β-sheets, nine α-helices and a reactive central loop to capture target proteases [23]. In a recent survey of the Central-European population, the average serum concentration of A1AT was 1.3 mg/mL [24]. Antitrypsin levels can increase in response to illness, infections or stress. In exacerbations of COPD, antitrypsin levels may double.

A1AT mutations as a cause of emphysema were discovered in 1963 [25], and since then, exogenous antitrypsin (augmentation therapy) has become available to compensate for defective gene products [26]. Considering its broad range of biological functions, antitrypsin therapy can bring new possibilities in many areas. The occasional dosing of A1AT in critically ill patients with infections may mitigate consequences leading to emphysema [27] and symptoms of CF patients with elevated neutrophil elastase concentrations that cause the deterioration of the airway tissue [28,29].

When basing disease recognition and therapy on smokers with rapidly declining lung function and low serum A1AT levels, A1AT deficiency is still an under-diagnosed condition [30], although cost-effective real-time PCR-based methods are available to detect mutations among at-risk populations. The rate of major *SERPINA1* mutations can be as high as 4–15% in certain populations [31,32,33]. Identifying at-risk smokers by A1AT genotyping combined with specific and timely intervention to prevent the deterioration of their condition would reduce health care costs. The role of antitrypsin in lung diseases other than COPD remains elusive, as its levels are rarely monitored in clinical practice, though such patients may also benefit from genotyping and appropriate treatment.

The need to assess the risks for patients heterozygous for *SERPINA1* mutations has also been raised [34], even though their antitrypsin levels may not fall below the normal range and they may not develop fulminant COPD as homozygotes do. Finally, in contrast to the better-known Z and S mutations reviewed recently by Hernandez [35], little attention has been paid to date to the ever-increasing number of rare mutations [36].

Although the role of antitrypsin in lung diseases other than COPD has started to be recognized [37,38,39,40,41,42,43,44,45], it is still far from well-known.

Antitrypsin irreversibly binds target proteases to its reactive center loop, thereby inactivating the protease. However, the antiprotease inhibitory activity of antitrypsin is tightly regulated by the oxidation of the sulfur-containing amino acid methionine. ROS and NOS species can both react with antitrypsin converting methionine to methionine sulfoxide residues, resulting in the inactivation of the enzyme [19]. This way, A1AT inactivated by oxidants loses its protease-neutralizing ability, leading to neutrophil elastase degradation in lung tissue. ROS and NOS species may originate from external sources such as smoke or air pollution, but intrinsic ROS production is also a significant factor in various diseases and infections. ROS stemming from other sources can also inactivate antitrypsin, leading to enhanced tissue destruction.

In this study, we aimed to investigate the impact of major A1AT mutations in several lung pathologies, given the risks that may exist and the potential benefits of augmentation treatment in the long term or in acute events. In addition to mutations in the coding region, variants in the quantitative trait loci (eQTLs) that are thought to modulate A1AT concentrations were also investigated. The effect of smoke on A1AT levels has been explored in lung diseases other than COPD, as they may exacerbate the underlying disease. This study also aimed to shed light on the possible involvement of A1AT in the development of comorbidities. Cardiovascular comorbidities and COPD frequently occur together and share similar pathophysiological mechanisms [46,47]. One such example is the reciprocal relationship between atherogenic disease and COPD [48,49], where increased intrinsic ROS production leading to atherogenic remodeling also enhances COPD, whereas increased oxidized LDL production by smoke enhances atherogenesis [50,51]. In addition to serum levels, the elastase activity of antitrypsin was also assessed, as it is directly linked to its ability to counteract the destructive effects of oxidants. Finally, A1AT variants and protein levels were correlated with clinical and respiratory parameters.

## 2. Results

### 2.1. Groupwise Differences in Blood A1AT Levels

**Figure 1:**
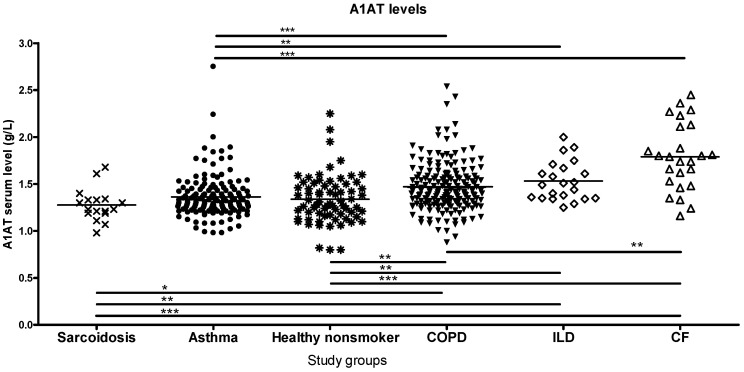
Serum alpha1-antitrypsin (A1AT) levels in various lung pathologies. COPD, ILD and CF groups have a significantly increased antitrypsin level compared to control, asthma and sarcoidosis groups. Patients with CF had the highest while patients with sarcoidosis had the lowest antitrypsin levels. (significant relations by ANOVA: * *p* < 0.05, ** *p* < 0.01, *** *p* < 0.001).

Lung diseases can be ranked on an ascending scale based on serum antitrypsin levels (Figure 1).

Patients with sarcoidosis were found to have the lowest levels of serum A1AT, lower than healthy controls. A1AT levels were not different in patients with asthma compared to healthy subjects but were significantly elevated in patients with COPD, ILD and CF. While smokers without COPD presented only a small but not significant increase with respect to non-smoker controls, the smoker and smoker with COPD groups were significantly different in antitrypsin levels (Figure 1, Supplementary Table S1).

**Figure 2:**
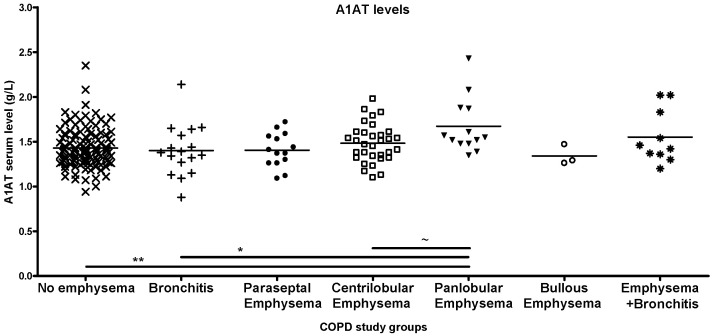
Distribution of A1AT levels in various COPD phenotypes based on findings of low-dose computer tomography (LDCT). Alpha1-antitrypsin (A1AT) levels show significant differences depending on the presence and type of emphysema. COPD patients with panlobular emphysema have higher levels of serum A1AT than those without emphysema or with bronchitis. The difference between centrilobular and panlobular emphysema is only of borderline significance (significant relations by ANOVA: * *p* < 0.05, ** *p* < 0.01, ~ *p* < 0.055).

COPD subgroups,- such as patients with no emphysema, patients with bronchitis or with paraseptal or bullous emphysema (as established by LDCT scans),- presented similar levels of antitrypsin, while those with centrilobular emphysema and panlobular emphysema had higher levels. The co-occurrence of emphysema and bronchitis was also associated with higher antitrypsin levels (Figure 2).

Antitrypsin increased with severity stages (GOLD 1-4) in COPD, while lung function decreased (Supplementary Table S1).

**Figure 3:**
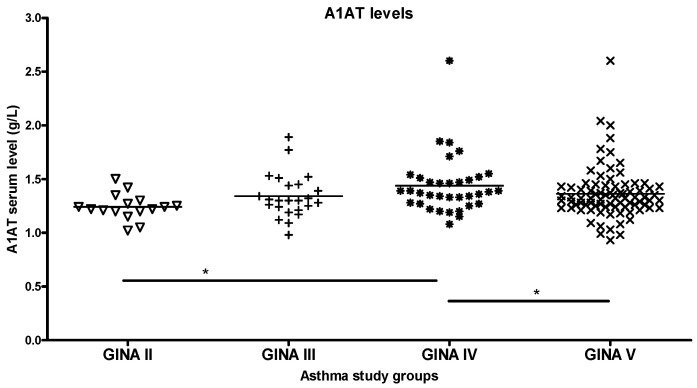
Serum alpha1-antitrypsin (A1AT) levels in patients with asthma stratified according to GINA. A1AT levels increase with the severity of asthma in all but the GINA V group (significant relations by ANOVA: * *p* < 0.05).

In patients with asthma classified by GINA, antitrypsin levels were elevated up to grade IV, while the GINA V group had lower levels (Figure 3).

**Figure 4:**
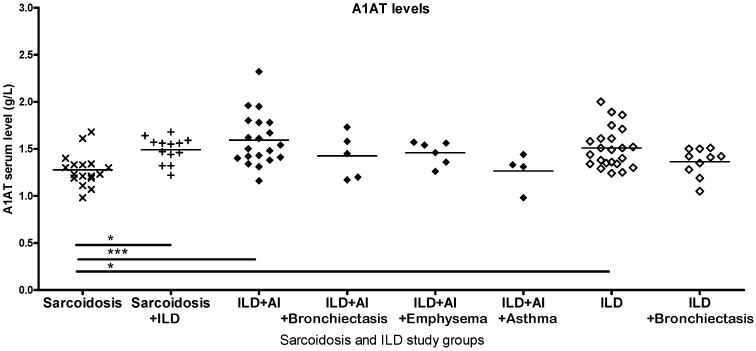
Serum alpha1-antitrypsin (A1AT) in sarcoidosis and ILD patient subgroups. AI = autoimmune disease. Significant differences in serum A1AT levels are present between sarcoidosis and ILD, and between sarcoidosis and ILD subgroups with autoimmune features (significant relations by ANOVA: * *p* < 0.05, *** *p* < 0.001).

Patients with ILD were stratified by accompanying diseases, as these can significantly alter the underlying disease. ILD subgroups had varying antitrypsin levels; however, the sample numbers were too low for comparisons. Most patients with ILD were smokers, with no difference between smoker and non-smoker groups. The co-occurrence of sarcoidosis and ILD results in an antitrypsin level similar to ILD alone (Figure 4). CRP and lung function values corresponding to each study group are listed in Supplementary Table S1.

Regarding the comorbidities detected on CT scans, current smokers and ex-smokers with COPD had significantly higher rates of a sclerotic aorta compared to age-matched individuals who had never smoked (*p* < 0.001). Coronary sclerosis was significantly higher in ex-smokers with COPD (*p* < 0.01). Atherosclerosis was prominent in non-smoker healthy controls and patients with ILD (Supplementary Table S2). No CT scans were available for all other patient groups.

Patient questionnaires revealed higher rates of hypertension in the smoker and ex- smoker groups compared to those who had never smoked. The overall rate of hypertension was higher in smokers. Hypertension was significantly increased in the smoker (*p* < 0.01), ex-smoker (*p* < 0.05) and COPD groups (and *p* < 0.05) compared to controls, while the rate of cardiovascular disease was increased in ex-smokers with COPD with borderline significance and smokers with ILD (Supplementary Table S2).

Looking at A1AT levels in more detail, antitrypsin was higher in comorbid patients with a sclerotic aorta, coronary sclerosis, atherosclerosis, cardiomegaly, cardiovascular diseases, skeleton disorders or liver disease than in non-comorbid patients. Lung functions and CRP values were similar in smoker groups with or without comorbidity.

In contrast, A1AT levels were similar in patients with and without hypertension, except in the ex-smoker COPD subgroup without hypertension, which had significantly higher levels of antitrypsin.

Serum lipid levels were similar between comorbid with sclerotic aorta, coronary sclerosis and atherosclerosis and non-comorbid patients. In contrast, lipid levels showed significant differences in smokers with hypertension and/or diabetes versus those without these comorbidities (Supplementary Table S3).

### 2.2. Differences by Genotype

Genotypic distribution was not significantly different for the S, Z, M and eQTL variants between groups (Supplementary Table S4). There were no 0 mutants in the whole study population. The frequency of the heterozygote S genotype was 2.7%, while that of the heterozygote Z genotype and homozygote S genotype was 1.7% and 0.09%, respectively. However, the study population was not randomly selected.

**Figure 5:**
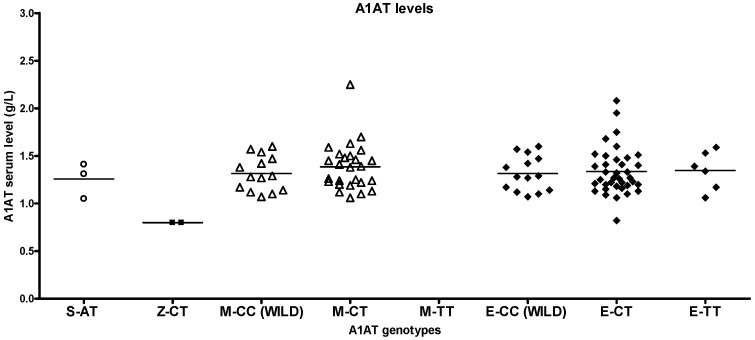
Serum alpha1-antitrypsin (A1AT) levels in healthy non-smokers with different A1AT genotypes. A1AT levels in S heterozygotes are in the normal range (0.89–2.05 g/L), while those in Z heterozygotes are low. Differences in antitrypsin levels in the case of M and eQTL variants were not significant.

**Figure 6:**
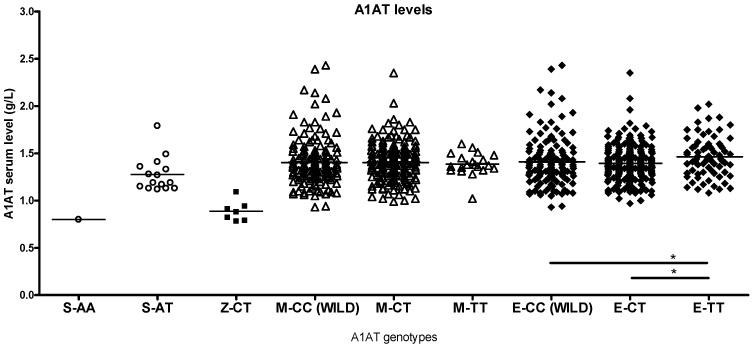
Serum alpha1-antitrypsin (A1AT) levels in smokers with different A1AT genotypes. The S-AA homozygote and Z-CT heterozygotes have antitrypsin levels below the normal range (0.89–2.05 g/L), while S-AT heterozygotes are in the normal range. All M genotypes have similar A1AT levels. Patients with the eQTL-TT genotype have significantly higher antitrypsin levels than other eQTL genotypes (significant relations by ANOVA: * *p* < 0.05).

**Figure 7:**
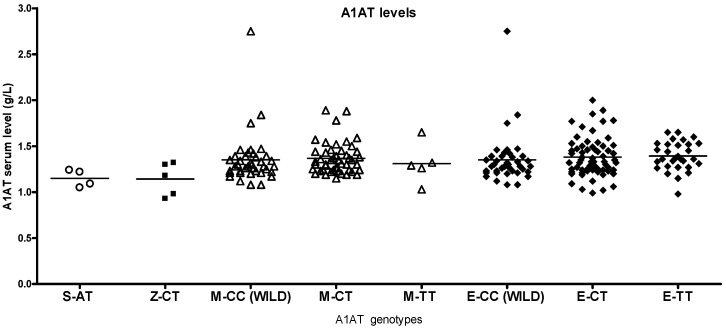
Serum alpha1-antitrypsin (A1AT) levels in patients with asthma with different A1AT genotypes. Z-heterozygote patients with asthma show higher while S heterozygotes have lower serum antitrypsin values compared to healthy controls and smokers. Differences in antitrypsin levels between M and eQTL variants were not significant.

S and Z heterozygotes in the study population had higher than normal antitrypsin levels (0.89–2.05 g/L), and most of the heterozygotes had normal lung function despite smoking (Supplementary Table S1). The corresponding other smoker genotypes had higher antitrypsin levels than healthy non-smokers or patients with asthma (Figure 5, Figure 6 and Figure 7).

**Figure 8:**
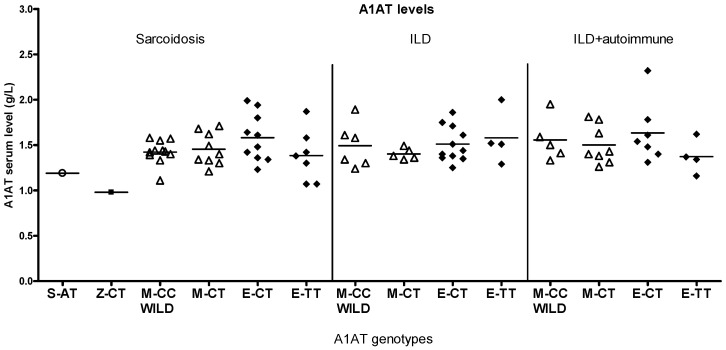
Serum alpha1-antitrypsin (A1AT) levels in ILD with different A1AT genotypes. While A1AT levels in M and eQTL variants appear different in sarcoidosis and ILD groups, these differences were not significan.In patients with asthma, Z heterozygotes had the lowest lung function values and they all belonged to the GINA V group with severe asthma; 4/5 of them were smokers (Figure 7, Supplementary Table S1). Their lung function values were even lower than those of Z-heterozygote smokers without asthma.

The TT genotype of the eQTL site had significantly higher antitrypsin levels compared to the other eQTL genotypes in smokers (Figure 6, Supplementary Table S1), with no significant reduction in lung function, while the E-TT genotypes in non-smokers and patients with asthma did not show increased antitrypsin levels. E genotypes in sarcoidosis and ILD + autoimmune groups showed some differences, but low case numbers impeded statistical analysis (Figure 8).

Although the number of patients with sarcoidosis and ILD per group is low, different antitrypsin levels were observed in each genotype (Figure 8).

**Figure 9:**
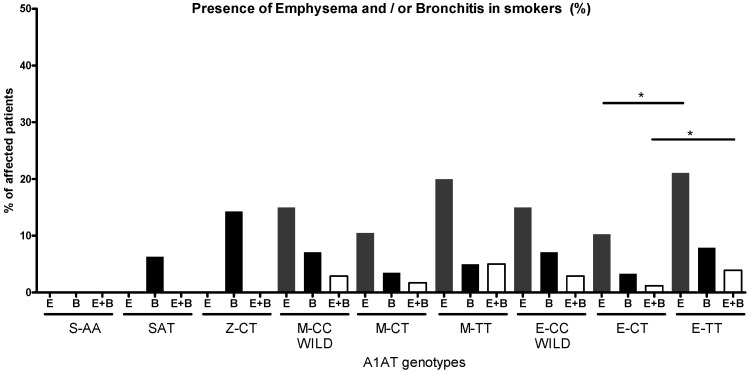
Presence (%) of emphysema and/or bronchitis among smokers with different A1AT genotypes. Emphysema is absent in S homozygote, S heterozygotes and Z heterozygotes. Bronchitis is significantly more prevalent in Z heterozygotes than in A1AT wild-type smokers. Smokers with the eQTL-TT genotype have significantly higher rates of emphysema (CT vs. TT *p* < 0.05) and a combination of emphysema and bronchitis compared to eQTL-CT genotypes (CT vs. TT *p* = 0.02). (significant relations by Chi2 test: * *p* < 0.05).

LDCT scans found no emphysema in S- and Z-heterozygote smokers; only bronchitis was present (Figure 9). Emphysema (CT vs. TT *p* < 0.05), bronchitis and a combination of these (CT vs. TT *p* = 0.02) were found more frequently in eQTL-TT genotypes than in CT genotypes.

In terms of comorbidity, some genotypes were more affected than others. Disease scores were lower in healthy non-smokers compared to smokers as based on LDCT and questionnaires data (Supplementary Table S4). Although genotype numbers are low, in S and Z heterozygotes, a higher incidence of cardiovascular features was observed (Supplementary Table S5).

In smokers, sclerotic aorta characterized the S heterozygotes; sclerotic aorta, coronary sclerosis and atherosclerosis were predominant in the M-TT genotypes, whereas coronary sclerosis was prevalent in the eQTL-TT genotypes, significancy levels were the following: *p* = 0.08 for E-CC vs. E-TT, and *p* = 0.04 for E-CT vs. E-TT (Supplementary Table S5). Hypertension rates were increased in smokers with M- and eQTL-variants—compared to non-smokers, though significance was not achieved, while smokers and non-smokers with S genotypes both had high rates. No diabetes cases were found among S heterozygotes, while Z-heterozygote smokers had high diabetes scores (M-CC (wild type) vs. Z-CT *p* = 0.0158, Supplementary Table S5).

Patients with asthma had lower comorbidity scores compared to smokers. Although the case numbers were low, Z heterozygotes with asthma had a high prevalence of hypertension, cardiovascular diseases and diabetes. eQTL-TT genotypes scored higher for hypertension (Supplementary Table S5).

The case numbers in the sarcoidosis, ILD and CF groups were too low to assess comorbidities by genotype. Certain rare risk genotypes and disease subgroups need more cases for statistical analysis.

### 2.3. A1AT Activity

**Figure 10:**
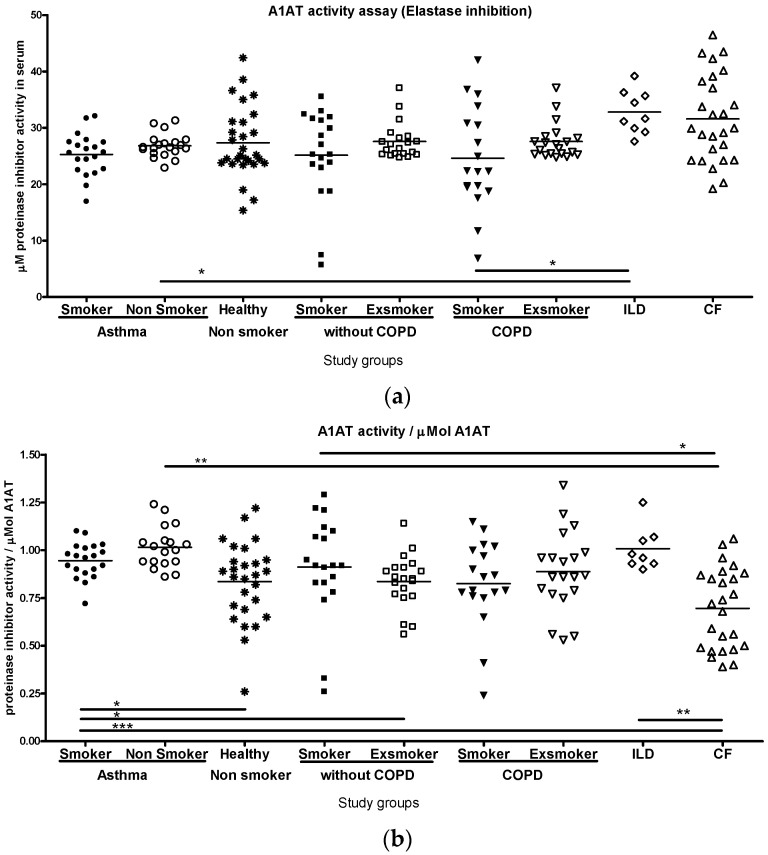
(**a**) A1AT antielastase activity in unit volume of serum in various lung pathologies. Healthy non-smoker controls, ex-smokers, ex-smokers with COPD and asthma patients all had similar unit volume activity, while the corresponding smoker groups exhibited lower activity. Antielastase activity was the highest in patients with ILD and CF (significant relations by ANOVA: * *p* < 0.05). (**b**) Serum A1AT molar activity levels (A1AT activity/A1AT concentration in μMol) in various study groups. Antitrypsin is more active in patients with asthma and ILD compared to healthy non-smoker controls, while activity is weakest in the CF group. A1AT activity is different between smokers with or without COPD (significant relations by ANOVA: * *p* < 0.05, ** *p* < 0.01, *** *p* < 0.001).

The antielastase activity of serum A1AT was similar in healthy non-smokers, ex-smokers and ex-smokers with COPD in unit volume of blood (Figure 10a), while in smokers, a decreasing trend was noted. Importantly, healthy smokers and smokers with COPD had similar antielastase activities despite different A1AT levels (Figure 1 and Figure 10a). Patients with ILD and CF displayed the highest antielastase activity.

Regarding molar activities of A1AT, patients with asthma and ILD displayed higher values compared to other groups, while patients with CF showed the lowest values. The molar activities in healthy smokers were higher than those of smokers with COPD (Figure 10b).

### 2.4. Correlations

**Figure 11:**
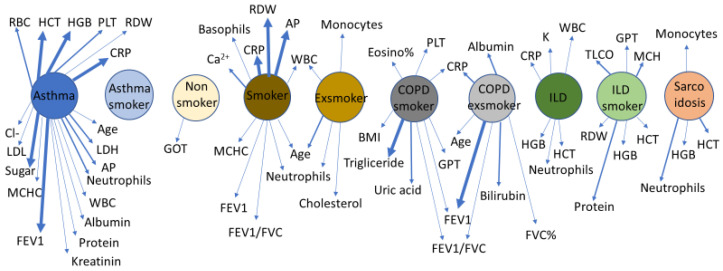
Correlation chart of alpha1-antitrypsin (A1AT). Clinically relevant serum parameters, as well as respiratory parameters, were correlated with antitrypsin levels by Spearman rank correlation in different lung pathologies. Only significant correlations are depicted in the chart (thicker lines mean stronger correlations, *p* < 0.05, *p* < 0.01, *p* < 0.001). While healthy non-smoker subjects have only one correlation with GOT values, several and diverse correlations characterized lung pathologies. Smoker groups show a different picture compared to non-smokers.

Glutamate oxaloacetate aspartate aminotransferase (GOT) was the only clinical laboratory parameter that correlated significantly with the level of A1AT in healthy non-smokers. In lung diseases, antitrypsin correlations varied between patient groups. Non-smoking patients with asthma showed the highest number of correlations, whereas in smokers with asthma, the A1AT level did not correlate with any blood parameters. ILD subgroups and sarcoidosis showed some similarities in terms of A1AT correlations. While A1AT levels did not correlate with FEV1/FVC in healthy non-smoker controls, it correlated inversely with FEV1 and FEV1/FVC in healthy smokers, smokers with COPD and ex-smokers with COPD. A1AT correlated directly with CRP levels in several patient groups, but in healthy non-smoker controls, smokers with asthma, smokers with ILD and patients with sarcoidosis, there was no correlation between A1AT and CRP (Figure 11). Smoking history (pack-years) was registered by self-report, and it did not correlate with any of the parameters.

## 3. Discussion

Our study suggests that A1AT plays a more important role in lung diseases than previously thought. Investigating a wide range of obstructive and interstitial lung diseases, we show that these conditions can be characterized by different A1AT levels. Antitrypsin levels increase in smoke-induced diseases, ILD and CF, compared to non-smoker controls, while at the other end of the scale, patients with sarcoidosis have lower antitrypsin levels. High antitrypsin levels may indicate ILD or CF. Low antitrypsin levels may be an indication not only of genetic antitrypsin deficiency but also of sarcoidosis or mild asthma. Alterations in antitrypsin levels are suggestive of damage to or hyperactivity of the antitrypsin molecule that is compensated by the modulation of its production, as suggested by this study. Furthermore, while almost no correlations between A1AT and clinical parameters were found in healthy, non-smoker controls, the number of associations increased with smoking and the presence of lung disease, implying the involvement of A1AT in several lung pathologies.

As for the genetic background of antitrypsin, there are over 500 different, genetically determined A1AT variants [36]. The three most important variants are types M, S and Z. The last two represent the most common abnormal variants that, when homozygous, are known to cause emphysema. The frequency of the Z allele is the highest in Baltic countries (4.5%), where this mutation probably originated, while in Eastern Europe, the frequency was estimated to be 1.0–1.5% in the study of Blanco et al. [31]. The S allele likely originated in the Iberian Peninsula, and a frequency of 15% has been reported in Spain by Hutchison [33]. Its frequency is estimated to be 2.5% in Hungary. The frequency of both mutants is far lower in Hungary than in the places where these alleles originate. In our study, the frequencies were close to the estimates, 1.7% for the Z allele and 2.7% for the S allele, but it is of note that this study was not a randomized population study.

Genetic testing for A1AT deficiency is recommended below a concentration of 1.2 g/L, and the ERS 2017 position paper defined 0.5 g/L as the limit of antitrypsin concentration that is thought to lead to emphysema [52]. In our study, patients carrying deficient alleles did not attain this low level, consistent with the observation that they did not develop emphysema. In our cohort, A1AT concentrations for Z heterozygotes were around 0.8 g/L, while the mean for S heterozygotes was around 1.2 g/L; the only S homozygote had a concentration of 0.8 g/L. These values are slightly lower than the values described by Stoller et al. [53], where the levels of Z and S heterozygotes and S homozygotes were 0.9 g/L, 1.25 g/L and 0.95 g/L, respectively. Antitrypsin levels decrease with age, with antitrypsin levels above 1.4 g/L in 25-year-old controls (unpublished results) and a mean of 1.34 g/L in patients over 50 years of age, so different ages may account for differences in concentrations.

Although deficient *SERPINA1* alleles resulted in reduced antitrypsin levels, there was no significant difference in the S, Z, M and eQTL distribution between lung pathologies. This means that most of these variants are not associated with any specific lung diseases, only the well-known S and Z homozygotes lead to severe COPD and emphysema in smokers. The modifying effect of S and Z may be limited to certain pathologic conditions, such as exposure to smoke and other processes involving oxidative stress. In asthma, Z heterozygotes appeared only in GINA V subgroup; however, case numbers were too low for a significant difference.

While antitrypsin deficiency is well known as a cause of emphysema, the effect of antitrypsin on other lung diseases is more obscure. A1AT levels were dependent on the severity and the type of emphysema in COPD. In smokers, increasing antitrypsin levels were associated with lower lung function values. A1AT levels increased with the severity grade in asthma, the etiology of ILD and the smoking status of subjects.

In our study, serum A1AT levels and lung function values were similar in heterozygote non-smokers and smokers carrying deficient alleles. Heterozygotes could compensate for the loss of function caused by a deficient allele. The survival of deleterious mutations is usually justified by some evolutionary advantage of the heterozygote form. This finding is confirmed by North et al. [54], who found that Z in heterozygote form increased both body height and lung function, and they suspected the Z allele to be a balanced polymorphism. While emphysema was absent in heterozygotes, bronchitis was more prominent. Comorbidities such as hypertension and cardiovascular disease were more common in S heterozygotes, while diabetes was more prevalent in Z heterozygotes, which makes knowledge of the exact genotype important in patient care. The role of A1AT in atherosclerosis, obesity and cardiovascular events is well documented [5,49,55,56,57,58,59,60]; however, distinct A1AT haplotypes or other modifying factors may have aggravating or alleviating effects, which may partly explain the conflicting findings of these studies.

In certain types of emphysema, antitrypsin levels are elevated, in parallel with CRP levels, implying greater systemic inflammation, which translates to reduced antioxidant capacity and worse lung functions. Nonemphysematous smokers had elevated CRP levels and worse lung function compared to emphysema patients with similar levels of antitrypsin. These patients are characterized by a predominance of airway disease fundamentally different from emphysema types, which explains the different behavior of this group.

A1AT levels in patients with CF have been studied previously, as massive neutrophil elastase (NE) concentrations have brought A1AT into focus. One of the main factors influencing antitrypsin in patients with CF is the increase in NE concentrations caused by the response to intense bacterial presence; an increase in antitrypsin may counteract this NE imbalance. Second, persistent bacterial or viral infections also cause an imbalance in oxidant status, which may compromise antitrypsin activity, which in turn is compensated for by an increase in antitrypsin production in patients with CF. Similarly to us, Frangolias et al. [41] found increased antitrypsin levels in patients with CF (1.73 g/L in patients with mild and 1.9 g/L in patients with severe CF), but antitrypsin levels in heterozygotes were not reported. Compared with our own and Frangolias’ studies, Amati et al. [42] found lower antitrypsin levels in CF patients with wild-type antitrypsin, but this could be explained by differences in CFTR gene mutations, levels of microbial colonization, antimicrobial treatments and medications. These factors may all influence antitrypsin levels. Döring et al. [43] found an earlier onset of *Pseudomonas* infection in CF patients with antitrypsin deficiency. These results and the high neutrophil elastase levels suggest a role for antitrypsin in the course of CF disease, but the prevalence of deficient alleles in patients with CF was not different from that in healthy individuals.

In patients with asthma, A1AT levels were lower in the case of S and higher in the case of Z heterozygotes, the opposite of those found in both the healthy non-smoker and smoker groups, close to normal antitrypsin levels. In accordance with our findings, a recent asthma study [37] found similar antitrypsin levels in wild-type patients and controls, where heterozygotes had a lower value, but no separate values were published for S and Z alleles. Deficient alleles were significantly increased in their asthma group, while this is only true for the GINA V subgroup in our study. The incidence of deficient alleles was higher and deficient A1AT concentrations were lower (0.78 g/L) compared to our results. These findings confirm that deficient *SERPINA1* alleles are risk factors for asthma. Other studies have also confirmed the involvement of antitrypsin in asthma and its exacerbations [44,45,61]. In our study, asthma was aggravated by smoke exposure in Z-heterozygote patients. The affected individuals had low lung function values, lower than those of smokers or patients with ILD, and all developed severe asthma. Siri et al. [62] and Stirpe and Bardaro [63] expressed doubts about the classification of these patients, mentioning common clinical features across asthma and A1AT deficiency. De Luca et al. [64] found an association of Z mutants with developing childhood asthma. Z-variant antitrypsin has an increased tendency for hydrophobic interactions, and Z-A1AT–fatty acid polymers strongly enhance *ANGPT14* expression and exacerbate inflammation-induced lung damage [65]. Cigarette smoke, on the other hand, can induce oxidative modifications of the Z-A1AT protein [66]. The numerous correlations between clinical parameters and A1AT in patients with asthma imply that the effect is even more pronounced in the case of a defective A1AT molecule. Antitrypsin levels increased with asthma severity. While in smokers, antitrypsin is expected to increase to replace molecules inactivated by smoke-induced ROS and NOS, in patients with asthma, the observation cannot be explained by the effect of smoke. However, oxidative stress may also be associated with asthma, as allergic and inflammatory reactions in patients with asthma could also contribute to the oxidant imbalance that impairs antitrypsin activity. The systemic inflammation present in patients with asthma was reflected by CRP levels, which also increased with severity (except in the GINA V group), and antitrypsin levels seemed to follow the CRP pattern. CRP is a marker of systemic inflammation [67].

A different picture of antitrypsin is presented by looking at the effect of mutations on antitrypsin levels and lung function. S, Z and M variants have missense mutations located in the coding region, with an impact on the structure of antitrypsin. Smoking appears to have little effect on these variants’ antitrypsin levels or lung function. However, the presence of asthma has a modifying effect on A1AT levels in S and Z heterozygotes and M-TT variants. Lung function was decreased in Z heterozygotes. Cases with sarcoidosis and ILD carrying the M-CT variant showed altered A1AT levels, but lung function values were similar for these variants, although case numbers were low. Different underlying diseases with different inflammatory pathways and oxidant status may influence the manifestation of different genotypes. Different genotypes may perform better or worse in different diseases, which is a matter that demands further exploration. Mutations in the coding sequence of A1AT may influence oxidative capacities and the depletion of antitrypsin activity in various lung pathologies.

The regulatory region eQTL only affects the amount of A1AT, not the protein structure. We hypothesize that it may be responsible for the modulation of antitrypsin levels, and a reduced availability of antitrypsin may underlie the adverse consequences in its variants. In smokers with the eQTL-TT genotype, antitrypsin levels increased, probably to compensate for the loss of activity. Smoking was associated with inferior clinical parameters, i.e., an increased incidence of emphysema and bronchitis alone and in combination. Therefore, the eQTL TT genotype can be considered a new disease-aggravating polymorphism, a possibility that might be worth evaluating further. Our study is the first to evaluate the influence of this eQTL genotype on the levels of antitrypsin and comorbid features. In asthma, eQTL variants have no effect, whereas in sarcoidosis and ILD, eQTL variants express different levels of antitrypsin. These results suggest that underlying diseases and interactions with different pathways have a modifying effect in these diseases.

A1AT has not yet been implicated in sarcoidosis or ILD, nor has the effect of the eQTL site been investigated. To date, no attention has been paid to different genotypes in terms of antitrypsin levels and comorbidities.

We hypothesize that a homeostatic compensatory mechanism is likely to underlie altered antitrypsin levels, ensuring that total antielastase activity is kept constant as molar activity decreases under a variety of conditions (smoke, stress, etc.). Reactive oxygen and nitrogen species generated by smoking lead to the inactivation of A1AT and a loss of its elastase-binding activity [19]. However, other activities of this multifaceted molecule are not affected by the inactivation of the elastase site [2,3]. Although smoking may not impair A1AT functions other than elastase activity, the resulting increase in antitrypsin may in itself induce other pathological conditions, as shown previously [5,68,69,70,71,72]. Smokers compared to controls had 10–20% higher rates of hypertension and higher rates of cardiovascular, diabetic, skeletal and thyroid disorders and tumors. The impact of smoking on metabolic syndrome is well known [73,74,75,76], and increased free valences of antitrypsin can serve as mediators that aggravate metabolic syndrome. Inouye found an increase in A1AT in atherosclerotic plaques and adipose tissue [77] and several associations with metabolic network metabolites, confirming a relation between antitrypsin levels and metabolic syndrome, components that our results also imply.

Our study demonstrated that A1AT levels are increased in pulmonary patients with atherogenic comorbidities compared to patients without such comorbidities. This increase was not due to increased systemic inflammation (CRP levels) and was not accompanied by differences in lung function. The increased A1AT levels were also not accompanied by alterations in lipid levels, as lipid levels rarely correlated with antitrypsin. Atherogenic processes induce endogenous ROS production, which impairs the antioxidant capacities of antitrypsin [48,49], and therefore, the body responds with increased antitrypsin production, as our studies on antitrypsin activity suggested. The impairment of A1AT activity by smoke and atherogenic processes is compounded, aggravating both diseases. In atherogenesis, LDL becomes oxidized, triggering a cascade that leads to cytokine release, which promotes inflammation and ROS production. ROS inactivate the antielastase activity of antitrypsin, while smoke directly oxidizes LDL, leading to enhanced atherogenesis [50,51]. Cardiovascular comorbidities and COPD share similar risk factors and pathophysiological mechanisms, with smoke contributing to the development of multimorbid diseases [73].

The presence or absence of hypertension does not lead to different antitrypsin levels, suggesting that hypertension and antitrypsin pathways are not directly linked. Rather, hypertension was more associated with serum lipids.

Finally, our results suggest that patients with COPD may require higher levels of A1AT than healthy smokers with a similar smoking history to provide comparable antielastase activity. Higher antitrypsin levels with decreased antielastase activity in COPD were confirmed by Milovanovic et al. [78]. This is also the case in the relation of ex-smokers with COPD versus healthy ex-smokers. This implies yet another mechanism of A1AT regulation, which might explain why some smokers develop COPD while others do not.

Limitations of our study include the participant recruitment system, which restricted the inclusion of more severe cases, as non-mobile patients with severe COPD are less willing to participate in screening. More than a thousand participants were enrolled in this study, but due to the lower mutation rates in our population, the numbers in certain groups were still below statistical power. The study was not randomized and therefore of limited value for population frequency estimates. High CRP values may have introduced potential bias, but if only patients with low CRP values are included, the differences in antitrypsin levels still hold. Steroid treatment is also a confounding factor. In this study, steroid users had similar or higher antitrypsin levels compared to steroid-naïve patients. Antitrypsin is only one of several contributors to the complexity of COPD.

In conclusion, alterations in antitrypsin levels and the underlying changes in antitrypsin activity could be important contributing factors in several lung diseases. Our results and those of others suggest that A1AT, in addition to its role in lung disease, is also involved in cardiovascular comorbidities and that these diseases may worsen each other.

## 4. Materials and Methods

### 4.1. Study Subjects

Blood samples were collected from healthy non-smokers (N^0^ = 85), healthy smokers (N^0^ = 291), healthy ex-smokers (N^0^ = 127), smokers with COPD (N^0^= 187) and ex-smokers with COPD (N^0^ = 64) above the age of 50 years, connected to a low-dose computer tomography (LDCT) study for lung cancer screening [79]. Blood samples from patients with asthma (N^0^ = 194), interstitial lung disease (ILD, N^0^= 93), sarcoidosis N^0^= 29) and cystic fibrosis (CF, N^0^ = 26) were collected during their scheduled ambulatory visits to our hospital (Table 1).

Only treatment-naïve patients with sarcoidosis and CFTR modulator-naïve patients with CF were selected for the study. All patients were in a clinically stable state with no signs of acute infection/exacerbation. Sample fractions were separated, and the supernatant and cell fractions were stored at −80 °C until processing. The diagnosis of COPD, asthma, sarcoidosis, ILD and CF was established by chest physicians, according to international guidelines (www.goldcopd.org (accessed on 25 March 2024), www.ginasthma.org (accessed on 22 November 2023), [80,81,82,83]). Clinical and respiratory parameters and A1AT levels were examined by routine laboratory tests. LDCT scans were evaluated for the presence of emphysema and comorbidities; however, patients with lung cancer were excluded. Participants completed questionnaires on their smoking history, comorbidities and medications. The research protocol was approved by the National Public Health Center (NNK 52792-5/2021/EÜIG), and all subjects gave written informed consent to participate in the study. All procedures performed in the study involving human participants were in accordance with the 1964 Helsinki Declaration and its later amendments.

### 4.2. Low-Dose Computer Tomography

Low-dose computer tomography (LDCT) recordings were performed by a Siemens Somatom Definition Edge CT (Munich, Germany). Syngo Via software (version VB60A_HF02) was used, including Pulmo 3D, and MM reading applications (Siemens, Munich, Germany). CT scans were evaluated by two independent radiologists for the presence of tumors, inflammatory residuals, emphysema, chronic bronchitis, interstitial lung disease (ILD), bronchiectasis, pleural-, pericardial-, cardiac- and lymph node alterations, atherosclerosis, coronary sclerosis, sclerotic aorta, calcifications, liver alterations, hernia and goiter. CT scans were also evaluated for the percentage of hypodense areas under −950 HU (Hunsfeld Unit) by the CT program. Lung was considered emphysematous above 6% of pulmonary volume under −950 HU density, using the method described by Mascalchi et al. [84].

### 4.3. DNA Isolation

Blood samples were separated from the supernatant, and cells were subjected to DNA isolation (Zymo Research DirectZol DNA/RNA Miniprep kit, (Zymo Research Corp, Tustin, CA, USA) and subsequent genotyping analysis. DNA extraction was performed from a 300 µL blood cell fraction according to protocol. DNA concentrations were measured by a Qubit fluorometer (Life Technologies, Foster City, CA, USA).

### 4.4. Real-Time PCR

Genotyping real-time PCR (qPCR) reactions were carried out with 2 µL DNA sample on an ABI 7500 machine (Life Technologies) in a total reaction mixture of 20 µL containing 10 µL Taqpath Proamp Mastermix (Life Technologies), 7 µL molecular-grade water and 1 µL Taqman SNP assay (Life Technologies) for *SERPINA1* (A1AT) at the following sites: S (rs17580), Z (rs28929474), M2/M4 (M) (rs709932), 0 (rs28929473) and eQTL (E) (rs2854254) [36]. Amplification in qPCR assays was performed with the following protocol: prereading phase at 60 °C for 30 s and initial denaturation at 95 °C for 5 min, followed by 40 cycles (95 °C for 15 s, 60 °C for 60 s). Genotyping assays were performed in two replicates; in case of discord, a third replicate was performed. The software instrument automatically identified genotypes dependent on fluorescence intensities of VIC and FAM reporter dyes.

### 4.5. A1AT Activity Assay

A1AT activity was estimated by measuring the inhibition of elastase activity of the serum according to the manufacturer’s protocol (Northwest Life Sciences LLC, Vancouver, BC, Canada). Briefly, samples were diluted 1:150, and absorbance was measured at 410 nm. Elastase calibration and data analysis were performed by a 2nd-order polynomial curve fitting.

### 4.6. Statistical Analysis

Data are presented as mean ± SEM or median with interquartile range, as appropriate. Data distribution was analyzed by the Kolmogorov–Smirnov test. Gene levels as well as clinical parameters were compared using either an unpaired Student’s *t*-test (parametric data) or the Mann–Whitney test (non-parametric data). Multiple-comparison analysis was performed using an ANOVA test. Correlation coefficients were calculated by Spearman’s method. Calculations were performed by GraphPad Prism 4.0 (GraphPad Software Inc., San Diego, CA, USA). A *p* value < 0.05 was considered significant.

Groupwise differences were calculated by logistic regression and Chi^2^-probe. The genotypic distribution and the allelic frequencies between the sample groups (healthy non-smokers, smokers (without COPD), ex-smokers (without COPD), smokers with COPD, ex-smokers with COPD, asthma, ILD, cystic fibrosis) were calculated by the Cochrane–Armitage test, and the deviation of the observed genotypes from the Hardy–Weinberg equilibrium was tested, with *p* values < 0.05 being accepted as significant. Post hoc power calculation for A1AT levels among the groups was performed with α = 0.05. Calculation was performed by the G*Power 3.1.1 (G*Power Software Inc., Kiel, Germany) software package.

## Figures and Tables

**Table 1:** Demographic data.

Study Groups	Patient Number	Male/Female Ratio	Age (Year ± SD)
Controls/healthy non-smokers	85	26/49	64.6 ± 10.7
Smokers (no COPD)	291	97/128	60.6 ± 7.2
Ex-smokers (no COPD)	127	61/45	63.4 ± 7.6
Smokers with COPD	187	81/70	63.4 ± 6.2
Ex-smokers with COPD	64	24/24	66.9 ± 8.4
Not categorized	85	42/29	62.6 ± 7.3
Asthma	128	49/79	54.3 ± 14.3
Smokers with asthma	66	34/32	58.5 ± 11.6
Sarcoidosis	17	9/8	49.6 ± 10.6
Sarcoidosis + F	13	9/4	51.4 ± 10.4
ILD + autoimmune	22	4/18	62.4 ± 9.4
ILD + AI + bronchiectasis	7	2/5	66.4 ± 13.2
ILD + AI + emphysema	7	2/5	67.5 ± 9.7
ILD + AI + asthma	4	1/3	48.5 ± 19.3
ILD	19	11/8	65.2 ± 15.8
ILD + bronchiectasis	10	4/6	71.5 ± 6.5
Cystic fibrosis	26	13/13	33.6 ± 8.9

## Data Availability

Supporting data can be found in Supplementary Tables S1–S5.

## Supplementary Materials

The following supporting information can be downloaded at https://www.mdpi.com/article/10.3390/ijms26115400/s1.

## Abbreviations

A1AT, antitrypsin; CRP, C-reactive protein; FVC, forced vital capacity; FEV1, forced expiratory volume in one second; TLC, total lung capacity; TLCO, carbon monoxide transfer factor; KLCO, carbon monoxide diffusion coefficient; ICS, inhaled corticosteroid; COPD, chronic obstructive pulmonary disease; GOLD, global initiative for chronic obstructive pulmonary disease; GINA, global initiative for asthma; ILD, interstitial lung disease; AI, autoimmune disease; CF, cystic fibrosis.
